# Sex, Immigration, and Patterns of Access to Primary Care in Canada

**DOI:** 10.1007/s10903-023-01459-4

**Published:** 2023-03-04

**Authors:** Joseph M. Ssendikaddiwa, Shira Goldenberg, Nicole S. Berry, M. Ruth Lavergne

**Affiliations:** 1 Faculty of Health Sciences, Simon Fraser University, 8888, University Dr, Burnaby, BC V5C 1S6 Canada; 2 Division of Epidemiology and Biostatistics, San Diego State University, 5500 Campanile Drive, San Diego, CA 92182-4162 USA; 3 Department of Family Medicine, Dalhousie University, 1465 Brenton Street, Halifax, NS B3J3TA Canada; 4 Centre for Gender and Sexual Health Equity, University of British Columbia, Vancouver, BC Canada

**Keywords:** Immigration, Primary care, Access, Sex

## Abstract

Access to primary care is crucial to immigrant health and may be shaped by sex and gender, but research is limited and inconclusive. We identified measures that reflect access to primary care using 2015–2018 Canadian Community Health Survey data. We used multivariable logistic regression models to estimate adjusted odds of primary care access and to explore interaction effects between sex and immigration group (recent immigrant: < 10 years in Canada, long-term immigrant: 10 + years, non-immigrant). Recency of immigration and being male were negatively associated with access to primary care, with significantly lower odds of having a usual place for immediate care among male recent immigrants (AOR: 0.36, 95% CI 032–0.42). Interaction effects between immigration and sex were pronounced, especially for having a regular provider or place of care. Results underscore the need to examine approachability and acceptability of primary care services, especially for male recent immigrants.

## Background

Globally, primary care is crucial to shaping immigrants’ health. Primary care plays an essential role for immigrants’ navigation of healthcare and associated welfare services upon settlement in a host country [1]. A strong primary care system is also associated with population health equity and is a cornerstone for achieving the health-related Sustainable Development Goals [2, 3]. Similar to other countries, primary care is intended to be the first and main point of access to health care in Canada. Primary care provides support for continuity and coordination of care when specialist or hospital services are required [4]. While there are international efforts toward equitable access to primary care, there are persistent gaps in equitable access, especially among marginalized groups including immigrants, refugees and minoritized ethnic groups [5, 6]. In Canada, while there is universal coverage for primary care for permanent residents, inequitable patterns of access to primary care among immigrants are widely reported [7–9]. Factors shaping these patterns include, language and cost related barriers, waiting periods for insurance, lack of knowledge needed to navigate the health system and lack of culturally appropriate care [10–12].

Patterns of access to primary care among immigrants may also be further shaped by sex and gender [13–16]. Sex is a multidimensional biological construct that encompasses anatomy, physiology, genes and hormones, which together affect how one is labelled and treated in the world [17]. Biological and physiological differences across the sex continuum may cause variation in the nature and severity of health issues [18, 19]. Female people may be more predisposed to reproductive and childbirth-related health issues, as such more likely to need and use health care services [14]. Gender includes socially constructed roles that affect how people perceive themselves, their expressions, behavior, actions and interactions [16, 17]. In describing the literature, we use the language in the literature cited (male/female, or genders), though many do not distinguish between sex and gender in their analysis. While biological sex may shape need for health care, gender may have more impact on both healthcare need and patterns of access to primary care. Gender is shaped by social, structural, and systemic factors, that may affect both need for care and how people interact with health systems [13, 20–22].

Combined with immigration, gender may also shape one’s conceptualization of health and resultant health seeking practices [13, 14]. Social determinants such as level of education, occupation, economic, and immigration status combined with gender may lead to differences in health status, health care needs, as well as differential resources to access care [23–25].

Internationally, while there are various studies that have examined the impact of immigration on access to primary care [6, 26–28], there is limited comparative analysis of patterns of access to primary care among sex and immigration groups. Many more studies explored experiences of access to primary care among immigrants, refugees and undocumented women than men [29–33].

Specific to Canada, research on the interaction of sex with immigration and its impact on primary care access is still limited and inconclusive [13, 34–36]. A study by Degelman and Herman using cross sectional data from the 2011–2012 Canadian Community Health Survey, found that compared to male recent immigrants, female recent immigrants are more likely to use primary care services [37]. Odds of having a regular doctor were higher among female recent immigrants than male recent immigrants [37]. Patterns of access to primary care observed among recent immigrant men are reported to be similar to patterns among non-immigrant men [37]. Within existing studies, immigrant women are more likely to report barriers to accessing immediate and routine care than non-immigrant women [9, 13, 34, 37]. Overall, while women are reported to use health care services more than men in Canada [13, 21, 22], studies suggest their experience rate poorly compared to that of men [34, 35]. Where studies have examined gender or sex and immigration, they have not considered the process or cascade of healthcare access, with consideration for the stage at which barriers arise between immigration and gender/sex groups. This information is needed for service planning.

Research on the interaction of sex and immigration and its impact on patterns of access to primary care is diverse and inconclusive partly due to limited conceptualization of healthcare access. The framework proposed by Levesque et al. [38] outlines the following stages: perception of needs and desire for care, healthcare seeking, reaching and utilization [38]. Applying this framework, may provide opportunities for more nuanced examination of immigration and sex-based differences in patterns of access. The social construct of gender may vary by immigration given the diverse background and origin of immigrants. All together this interaction may contribute to gendered experiences of need and desire for care. For example, because of varying gender roles, men and women may differ in perception of symptoms and the evaluation of severity of illness [14, 15]. A study that examines illness orientation as a determinant for sex based differences in utilization of medical care in the United States, found that, compared to men, women score significantly higher interest and concern with health, and report more symptoms [14]. Women may be more ready to express need and desire for care than men [39]. With gender roles, women are also more frequently involved in child care, including arranging health care for their children which may increase health care visits and as a result shape their own perception of needs [39–41].

A study exploring the socio-cultural factors of gender roles in women’s health care utilization in Southwest Nigeria [42] observed that, due to dominant patriarchal cultures in some societies, women’s health care needs and decisions are predominantly decided by the man [42]. As such, gendered perception of needs and desire for care may vary across cultures among immigrants and as a result, influence patterns of access upon settlement in a new system.

We therefore aim to examine how sex and immigration interact to shape patterns of access to primary care in Canada, using secondary analysis of survey questions that reflect access to primary care. Conceptualization and measurement of access is informed by a framework by Levesque et al. [38], which distinguishes potential from realized access, starting from identification and perception of needs and desire for care, to how care is sought, reached, and ultimately used.

## Methods

### Data Collection

We used Canadian Community Health Survey (CCHS) data. The CCHS is an annual, cross sectional national survey conducted by Statistics Canada. It is a representative sample of 98% of the Canadian population age 12 years and older living in private dwellings, excluding those residing on reserves and other Aboriginal settlements, full-time members of the Canadian Forces, institutionalized populations, and those residing in remote regions [43]. The sampling frame is based on place of residence and may therefore include people residing in Canada temporarily as well as Canadian citizens and permanent residents. Data was accessed through Statistics Canada Public Use Microdata files [13]. We used 2015–2018 cycles pooled into single data set to maximize sample size for all population strata. The survey is instrumental as it provides current, detailed, and uniform information about health in every Canadian province and territory and includes a range of questions related to primary care access, immigration, and gender, including several newly added since 2015 [13].

### Participants/Respondents

The sample included respondents aged 18 and older. We excluded respondents missing data for immigrant status, sex and measures of primary health care use and access.

## Measures

### Explanatory Variables

Sex is the primary explanatory variable. The CCHS asks “is respondent male or female.” The variable is labeled sex. It is possible respondents may interpret this to mean sex assigned at birth, legal sex, or gender. “Immigrant group” was the other main explanatory variable. Respondents were grouped into recent immigrants (immigrants who have lived in Canada for 0–9 years), long-term immigrants (immigrants who have lived in Canada for more than 10 years), and non-immigrants (people born in Canadian).

We used Andersen’s model of health utilization [44] to guide the identification and choice of covariates that are associated with sex and migration and that may also shape patterns of access to care from the CCHS. Variables include resource-based characteristics that play a role in one’s ability to access or use health care. All explanatory variables and corresponding categories are summarised in Table 1 below.

**Table 1:** Explanatory variables adopted from the CCHS and the Andersen Model

Explanatory variables	Variable categories
Immigration group	Recent immigration
	Long-term immigration
	Non-immigrant (Canadian born)
Sex	Male
	Female
Age groups (years)	18–34
	35–54
	55–74
	75 +
Racialization	White
	Non-white
Sexual orientation	Heterosexual
	Gay/Bi-sexual
Province of Residence	The Maritimes (New Brunswick, Prince Edward Island and Nova Scotia)
	Quebec
	Ontario
	The Prairies (Manitoba, Saskatchewan, Alberta)
	The Territories
	British Columbia
Marital status	Married/common law
	Widowed/separated/divorced/single
Knowledge of official languages	English/French
	Neither English nor French
Personal income	Less than S39k
	$40k to $79k
	$80k +
Education	Less than secondary
	Secondary
	Post-secondary
Perceived health status	Excellent
	Very good
	Good
	Fair
	Poor

### Outcome Variables

We selected available outcome variables related to primary care services from the CCHS between 2015 and 2018. We use the Levesque framework [38] to consider stages of access and group variables in order of the framework, from perception of needs and desire for care, health care seeking, reaching and utilization. While available variables do not directly measure each stage, organizing them into stages of the Levesque framework helps more clearly identify where gaps arise for each immigration and gender group and compare patterns of access between groups.

The variable “No regular provider because of no need” reflects perception of needs and desire for care” it includes reasons for not having a regular health care provider either because respondent had no need for one, had a regular care provider or a reason other than having no need.

Two variables describe having a regular place of care. The first, “Usual place for immediate care for minor problem” reports whether or not a respondent had a usual place for immediate care for minor problems. The second, “Type of usual place for immediate care for minor problem” reports whether usual place of care was an emergency healthcare service or other.

“Waiting time for immediate care for minor problem” was selected to capture how long respondents reported waiting before reaching needed care. The last variable “Consulted with a Family Doctor or General Practitioner in the past 12 months” measures utilization of services from a primary care physician.

For all the above variables, survey responses including “Valid Skip” and “Don’t know” or “Missed” were excluded. All outcome variables and corresponding categorical responses are summarised in (Table 2) below.

**Table 2:** Outcome measures with corresponding categories grouped by stage in Levesque framework

Stage of access and measure	Categories
Perception of needs and desire for care	
No regular provider no need	Yes
	No
Seeking	
Has a usual place for immediate care for minor problem	Yes
	No
Type of usual place for immediate care for minor problem	Walk-in clinic/emergency room some other place
Reaching	
Waiting time for immediate care for minor problem	Same/next day appointment
	More than same/next day
Utilization	
Consulted with a family doctor or general practitioner	Yes
	No

### Analysis

We calculated unweighted frequencies and weighted percentages of all responses and explanatory variables by immigration and sex (Tables 3, 4). We used survey weights in the percentages to account for response rates across groups.

**Table 3:** Descriptive characteristics (unweighted counts and weighted percentages) of respondents stratified by immigration group and sex, CCHS 2015–2018

Characteristic	Male recent immigrant	Male long-term immigrant	Male non-immigrant	Female recent immigrant	Female long-term immigrant	Female non-immigrant	p-values (χ^2^)
	N = 3336 (2.9)	N = 11,039 (8.3)	N = 86,094 (38.2)	N = 3858 (3.2)	N = 12,691 (8.6)	N = 99,885 (38.9)	
	n (%)	n (%)	n (%)	n (%)	n (%)	n (%)	
Age group (years)							< 0.0001
18–34	1220 (40.6)	1152 (15.5)	17,742 (28.6)	1627 (43.8)	1221 (14.0)	19,973 (27.3)	
35–54	1466 (43.0)	3107 (35.4)	22,455 (29.7)	1599 (39.7)	3800 (37.1)	25,764 (28.9)	
55–74	176 (5.9)	4669 (36.8)	29,755 (27.2)	233 (8.3)	4982 (35.5)	34,095 (28.0)	
75 +	32 (1.0)	1896 (10.5)	8010 (5.9)	33(0.8)	2494 (11.8)	12,323 (7.6)	
Missing	442 (9.5)	215 (1.8)	8132 (8.7)	366 (7.5)	194 (1.6)	7730 (8.2)	
Racialization							
White	685 (19.8)	5881 (37.6)	75,628 (85.5)	677 (15.8)	6948 (39.4)	88,271 (86.0)	< 0.0001
Non-White	2610 (78.6)	5024 (61.2)	3960 (8.9)	3144 (83.3)	5561 (59.3)	4082 (8.4)	
Missing	41 (1.5)	134 (1.2)	6506 (5.6)	37 (1.0)	182 (1.2)	7532 (5.6)	
Sexual orientation							
Heterosexual	2879(87.5)	9980 (90.4)	75,777 (88.4)	3428 (89.2)	11,526 (89.0)	89,530 (88.9)	< 0.0001
Gay/Bisexual	107 (3.4)	215 (1.9)	2210 (2.9)	72 (2.2)	203 (1.5)	3055 (3.6)	
Missing	350 (9.1)	844 (7.7)	8107 (8.7)	358 (8.7)	962 (9.5)	7300 (7.5)	
Province of residence							
The Maritimes (NB, Newfoundland, PEI, NS)	93 (1.2)	231 (0.8)	11,451 (8.2)	94 (1.1)	271 (0.8)	14,049 (8.5)	< 0.0001
Quebec	597 (19.4)	1330 (13.5)	19,732 (25.6)	650 (18.0)	1378 (12.7)	22,379 (25.8)	
Ontario	862 (38.6)	4817 (54.7)	23,748 (34.5)	1022 (44.3)	5584 (55.4)	28,994 (34.8)	
The Prairies (Manitoba, Alberta, Saskatchewan)	603 (15.6)	2564 (17.7)	10,274 (12.2)	737 (13.4)	3004 (18.2)	11,834 (12.2)	
The Territories	28 (0.1)	125 (0.1)	2430 (0.3)	53 (0.1)	127 (0.1)	2553 (0.4)	< 0.0001
British Columbia	1153 (25.2)	1972 (13.2)	18,459 (19.0)	1302 (23.1)	2327 (12.9)	20,976 (18.4)	
Marital status							
Married/common-law	2027 (64.8)	7320 (72.8)	44,324 (56.6)	2546 (68.0)	6685 (62.3)	47,157 (53.5)	< 0.0001
Widowed/Separated/divorced/Single	1305 (35.2)	3672 (26.9)	41,587 (43.2)	1301 (31.8)	5943 (37.3)	52,490 (46.3)	
Missing	4 (0.1)	47 (0.3)	183 (0.2)	11 (0.2)	63 (0.3)	238 (0.2)	
Knowledge of official language (English/French)							
English/French	3320 (96.2)	10,822 (97.4)	85,934 (99.7)	3634 (92.6)	12,229 (95.2)	99,732 (99.8)	< 0.0001
Neither English nor French	110 (3.6)	210 (2.6)	88 (0.2)	221 (7.4)	445 (4.7)	95 (0.1)	
Missing	6 (0.2)	7 (0.0)	72 (0.1)	3 (0.1)	17 (0.2)	58 (0.1)	
Personal income							
Less than $39k	1662 (55.4)	5042 (48.2)	35,493 (40.8)	2695 (74.1)	7686 (60.7)	57,670 (56.6)	< 0.0001
$40k–$79k	773 (22.5)	3361 (28.7)	25,433 (28.9)	538 (11.7)	3073 (23.3)	23,295 (23.1)	
$80k +	388 (9.9)	2099 (17.9)	15,292 (19.3)	152 (3.2)	1289 (9.7)	8874 (9.4)	
Missing	513 (12.2)	537 (5.2)	9876 (11.0)	473 (11.0)	643 (6.4)	10,046 (10.8)	
Education							
Less than secondary	555 (13.6)	1376 (10.4)	21,210 (20.1)	570 (14.4)	1893 (13.1)	22,059 (18.5)	< 0.0001
Secondary	511 (18.4)	2041 (19.5)	19,042 (23.4)	569 (15.9)	2616 (20.5)	22,250 (22.7)	
Post-secondary	2243 (67.3)	7432 (68.0)	44,802 (55.2)	2669 (68.3)	7949 (64.2)	54,366 (57.5)	
Missing	27 (0.6)	199 (2.0)	1040 (1.5)	50 (2.2)	233 (2.2)	1210 (1.4)	
Perceived health status							
Excellent	1199 (35.5)	2333 (22.7)	18,197 (24.4)	1207 (30.1)	2520 (20.6)	21,230 (24.0)	< 0.0001
Very good	1173 (34.3)	3615 (33.5)	31,146 (37.7)	1336 (33.9)	3988 (31.0)	37,441 (38.7)	
Good	806 (25.5)	3442 (31.5)	24,836 (27.2)	1092 (29.6)	4072 (33.2)	27,517 (26.1)	
Fair	121 (3.6)	1133 (8.7)	8566 (7.8)	172 (5.0)	1441 (10.2)	9890 (8.3)	
Poor	35 (1.1)	491 (3.5)	3204 (2.7)	46 (1.2)	630 (4.4)	3661 (2.9)	
Missing	2 (0.1)	25 (0.1)	145 (0.1)	5 (0.2)	40 (0.5)	146 (0.1)

**Table 4:** Unweighted counts, weighted percentages and p-values for primary care outcomes stratified by immigrantion group and sex, CCHS 2015–2018

Primary care outcome	Male recent immigrant	Male long-term immigrant	Male non-immigrant	Female recent immigrant	Female long-term immigrant	Female non-Immigrant	p-values (χ^2^)
	(N = 3336)	(N = 11,039)	(N = 86,094)	(N = 3858)	(N = 12,691)	(N = 99,885)	
	n (%)	n (%)	n (%)	n (%)	n (%)	n (%)	
No regular provider no need							
Yes	317 (9.8)	408 (3.9)	4665 (5.6)	191(4.7)	283 (2.4)	2690 (2.6)	< 0.0001
No	2978 (90.3)	10,603 (96.1)	80,902 (94.4)	3638 (95.3)	12,381 (97.6)	96,817 (97.4)	
Has usual place for immediate care for minor problem							
Yes	2615 (79.3)	9812 (88.5)	74,214 (85.7)	3256 (85.5)	11,641 (91.49)	91,330 (91.3)	< 0.0001
No	697 (20.7)	1186 (11.5)	11,540 (14.3)	584 (14.6)	1014 (8.5)	8195 (8.7)	
Type of usual place for immediate care for minor problem							
Walk-in clinic/ER	1231 (46.5)	2977 (31.0)	27,284 (39.1)	1424 (44.0)	3231 (30.1)	27,284 (32.4)	< 0.0001
Some other place	1374 (53.5)	6810 (69.0)	46,680 (60.9)	1825 (56.0)	8383 (60.9)	63,134 (67.6)	
Waiting time for Immediate care for minor problem							
Same/Next Day appointment	1092 (54.0)	4300 (51.6)	24,377 (40.8)	1398 (52.4)	5041 (48.3)	30,682 (40.0)	< .0001
More than Same/Next day	953 (46.0)	4649 (48.5)	39,049 (59.2)	1325 (47.6)	5812 (51.7)	51,883 (60.0)	
Consulted with a family doctor or general practitioner							
Yes	842 (55.5)	3770 (68.2)	27,040 (76.9)	1156 (66.0)	4733 (76.9)	36,767 (74.2)	< 0.0001
No	699 (44.5)	1513 (31.8)	15,062 (38.0)	612 (34.0)	1270 (23.1)	12,086 (25.8)	

*Source:* Canadian Community Health Survey, Statistics Canada

**Fig. 1:**
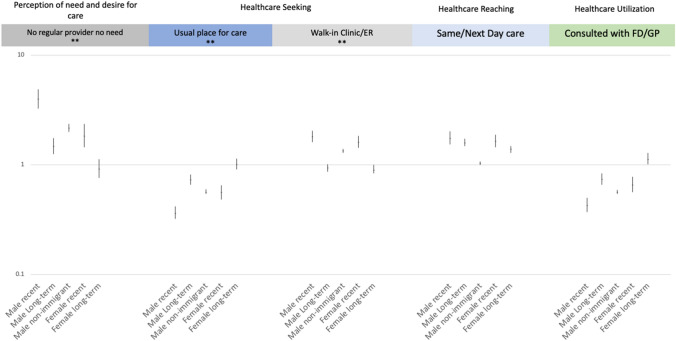
Log-scale graph showing adjusted odds estimates and 95% CI of sex and immigrant groups compared to female non-immigrants

We used logistic regression to examine the unadjusted association between immigration groups stratified by sex and primary care measures. Odds estimates for all immigration groups by sex are compared to female non-immigrant category given the highest response rate. (Table 5 and Fig. 1) We used multivariable logistic regression models to calculate adjusted associations between immigration and sex groups and all other covariates. We report adjusted odds ratios and 95% confidence intervals. Finally, we use logistic regression joint tests to calculate significance of interaction effects of immigration and sex with associated p-values (Table 5, Fig. 1). All statistical analysis was completed using SAS 9.4.

**Table 5:** Adjusted and unadjusted odds ratios for chosen primary care outcomes by immigration group and sex, p-values for tests of interaction effects, CCHS 2015–2018

Primary care outcome	Unadjusted estimates	Adjusted estimates	p-values (χ^2^)
	OR (95% CI)	OR (95% CI)	Immigration group * sex
No regular provider because of no need (Yes vs No) N = 215,873			
Male recent immigrant vs Female non-immigrant	4.00 (3.25–4.92)	3.95 (3.21–4.86)	0.0710
Male long-term immigrant vs Female non-immigrant	1.50 (1.26–1.77)	1.47 (1.24–1.75)	
Male non-immigrant vs Female non-immigrant	2.20 (2.02–2.39)	2.17 (2.00–2.35)	
Female recent immigrant vs Female non-immigrant	1.83 (1.44–2.32)	1.84 (1.44–2.35)	
Female long-term immigrant vs Female non-immigrant	0.92 (0.76–1.10)	0.92 (0.76–1.12)	
Usual place for immediate care for minor problem (Yes vs No) N = 216,084			
Male recent immigrant vs Female non-immigrant	0.37 (0.32–0.42)	0.36 (0.32–0.42)	0.0060
Male long-term immigrant vs Female non-immigrant	0.74 (0.66–0.82)	0.73 (0.66–0.82)	
Male non-immigrant vs Female non-immigrant	0.57 (0.54–0.60)	0.56 (0.54–0.60)	
Female recent immigrant vs Female non-immigrant	0.56 (0.49–0.65)	0.56 (0.48–0.65)	
Female long-term immigrant vs Female non-immigrant	1.03 (0.92–1.15)	1.01 (0.91–1.14)	
Type of usual place for immediate care for minor problem (Walk-in Clinic/ER vs Some other place) N = 192,312			
Male recent immigrant vs Female non-immigrant	1.82 (1.61–2.05)	1.81 (1.61–2.04)	< 0.0001
Male long-term immigrant vs Female non-immigrant	0.94 (0.87–1.01)	0.94 (0.87–1.01)	
Male non-immigrant vs Female non-immigrant	1.34 (1.29–1.39)	1.34 (1.29–1.39)	
Female recent immigrant vs Female non-immigrant	1.64 (1.46–1.85)	1.62 (1.43–1.83)	
Female long-term immigrant vs Female non-immigrant	0.98 (0.84–1.00)	0.90 (0.83–1.01)	
Same/Next Day for immediate care for minor problem (Same/Next Day vs More than same or next day) N = 170,561			
Male recent immigrant vs Female non-immigrant	1.76 (1.54–2.02)	1.76 (1.54–2.02)	0.1241
Male long-term immigrant vs Female non-immigrant	1.60 (1.48–1.72)	1.60 (1.45–1.87)	
Male non-immigrant vs Female non-immigrant	1.04 (1.00–1.10)	1.03 (1.00–1.07)	
Female recent immigrant vs Female non-immigrant	1.65 (1.46–1.87)	1.64 (1.45–1.87)	
Female long-term immigrant vs Female non-immigrant	1.41 (1.31–1.51)	1.39 (1.29–1.49)	
Consulted with a family doctor or general practitioner (Yes vs No) N = 105,550			
Male recent immigrant vs Female non-immigrant	0.43 (0.37–0.50)	0.43 (0.37–0.50)	0.1157
Male long-term immigrant vs Female non-immigrant	0.74 (0.67–0.83)	0.74 (0.66–0.83)	
Male non-immigrant vs Female non-immigrant	0.57 (0.54–0.60)	0.57 (0.54–0.60)	
Female recent immigrant vs Female non-immigrant	0.67 (0.58–0.79)	0.66 (0.56–0.78)	
Female long-term immigrant vs Female non-immigrant	1.15 (1.03–1.29)	1.13 (1.01–1.28)	

Source: Canadian Community Health Survey, Statistics Canada

Covariates in multivariable models used to generate adjusted estimates include age, racialization, sexual orientation, region of residence, marital status, knowledge of official language, personal income, education, and perceived health

Research study did not require ethics approval or ethical review as data was accessed through Statistics Canada Public Use Micro data files.

## Results

### Characteristics of Immigration Groups by Sex

Both male and female recent immigrants were younger than long-term and non-immigrant respondents, with a higher percentage of respondents below 34 years (40.6% among male recent immigrants, 43.8% among female recent immigrants) (Table 3). Higher percentages of immigrants identified as being non-White than non-immigrants. A slightly higher percentage of female non-immigrants and male recent immigrants identified as being gay or bisexual than other groups (3.6% and 3.4% respectively). A higher percentage of male long-term immigrants (72.8%) and both male and female recent immigrants (64.8% and 68.0% respectively) reported being married or in common law relationships than other immigration and sex groups. A lower percentage of male and female recent immigrants (96% and 92.6% respectively) and female long-term immigrants (95.6%) speak English/French compared to other groups (Table 3).

Across all immigration groups there was a higher percentage of female respondents in the lower income bracket ($39,000 per year) than males. Male recent immigrants were underrepresented in the middle ($40,000–79,000 per year) and high income (more than $80,000 per year) categories compared to male long-term and non-immigrants. A higher percentage of male and female recent immigrants followed by male and female long-term immigrants reported to have post-secondary education than non-immigrants (Table 3). A higher percentage of male and female recent immigrants (35.5% and 30.1% respectively) reported excellent to very good perceived health status compared to long-term and non-immigrants. Overall, while immigrants have higher education attainments than non-immigrants, immigrants are still overrepresented in the low-income category even after 10 years.

### Patterns of Access to Primary Care by Immigration and Sex

Where and how respondents sought primary care differed substantially among groups. Percentages of respondents reporting no usual place for immediate care were highest among male and female recent immigrants (20.7% and 14.6% respectively) followed by male non-immigrants and male long-term immigrants (14.3% and 11.5% respectively). Compared to female non-immigrants, odds of having a usual place for immediate care were lowest among male recent immigrants (AOR: 0.36, 95% CI 0.32–0.42) (Table 5, Fig. 1).

More male recent immigrants and male non-immigrants (9.8% and 5.6% respectively) followed by female recent immigrants (4.7%) reported no regular care provider because of no need (Table 4). Male recent immigrants and male non-immigrants had higher odds of having no regular provider because of no need compared to female non-immigrants (AOR male recent immigrants: 3.98, 95% CI 3.24–4.90, AOR male non-immigrants: 2.18, 95% CI 2.0–2.37).

A higher percentage of male and female recent immigrants (46.5% and 44% respectively) reported a walk-in clinic or emergency room as their usual place for immediate care followed by male non-immigrants (39.1%). Male and female recent immigrants had higher odds of using a walk-in clinic or emergency room for a usual place immediate care compared to female non-immigrant (AOR male recent immigrant:1.82, 95% CI 1.61–2.05), (AOR female recent immigrant:1.62, 95% CI 1.43–1.83) (Table 5, Fig. 1).

The percentage of male and female recent immigrants (54% and 52.4% respectively) who reported having same or next day appointments were slightly higher than long-term immigrants and non-immigrants (Table 4). Compared to female non-immigrants odds of having same or next day appointment were slightly higher among male and female recent immigrants (AOR male recent immigrants: 1.82, 95% CI 1.61–2.05), (AOR female recent immigrants: 1.62, 95% CI 1.43–1.83).

## Discussion

### Summary of main Results of the Study

Findings underscore significant interactions between immigration and sex in shaping patterns of access to primary care. Particularly, recency of immigration and identifying as male appears to coincide with more limited access across most measures. Overall, a higher percentage of male respondents across all immigration groups report lower access to primary care including having a regular place of care, usual place for immediate care for minor problem and consultations with a family doctor or general practitioner. Interaction effects of immigration and sex were significant for measures describing how respondents seek primary care services, but not for health care utilization (Table 5).

### Explanation of the Findings; Comparison and Contrast of Findings with Other Related Studies in the Literature), Policy Implications

Observed differences between immigration and sex groups appear to be more pronounced in patterns of how respondents perceive need and desire for care and how they seek primary care. This pattern may speak to how the interaction between immigration and gender roles shape perception and interpretation of symptoms or sickness among men and women, and resultant health care seeking patterns [14]. The higher percentage of male respondents reporting no regular provider because of no need and lower percentage of male respondents reporting a usual place for care, may be linked to performative gender roles and how they impact men’s perception and interpretation of illness [14]. Previous research also shows that due to gender roles, females show a higher interest and concern with health than males, which manifests in increased symptom reporting and desire for care [14, 24], and may subsequently affect utilization of health services [14].

Lower use of care among recent immigrants is sometimes attributed to the “healthy immigrant effect [12, 18].” Canadian immigration policies restrict entry to people who have health problems, and prioritize relatively people of working age, which is consistent with the observation that recent immigrants report better health status [18, 45, 46]. However, we attempt to adjust health status as part of need for health care, and disparities in access persist in adjusted models. For this reason, we believe findings suggest barriers to access.

As observed, while immigrants have higher education attainments than non-immigrants, immigrants are still overrepresented in the low-income category even after 10 years, which may reflect barriers to full recognition of credentials and experience outside of Canada [19, 47]. This may reinforce cost related barriers as recent immigrants are forced to prioritize employment and education over healthcare seeking to provide for their families and settlement expenses. Cost related barriers may also be linked to cultural and gendered roles between men and women which may provide plausible explanations for the significant differences in healthcare utilization and access patterns between recent immigrant men and women.

To some extent, observed differences between male and female recent and non-immigrants in their perception of need and desire for care may be shaped by recent immigrants’ expectations and previous structural experiences with health care systems including marginalization, discrimination, racism and language barriers [48–50]. Differences across systems may impact expectations and perceived need after settlement in Canada [51].

The observed high percentage of recent immigrants who report using walk-in clinics, emergency departments and same/next day care is consistent with previous research that has found utilization of walk-in clinics and same/next day care to reduce with increased length of stay in Canada as people find more regular sources of care such as family doctors [52]. Reported explanations for the use of walk-in clinics among recent immigrants may include convenience and flexibility of hours of operation given that walk-in clinics do not require appointment or scheduling. Flexibility and convenience are important among recent immigrants upon settlement in Canada [11, 53], because of competing needs such as employment, education and family settlement that may be prioritized over healthcare seeking in the first few years. These needs may also vary by sex or gender [54]. Use of walk-in clinics may also be linked to the sporadic development of need given the largely young immigrant population and may also reflect use of healthcare services consistent with place of origin [50]. This observation suggests a need to identify features of walk-in clinics that contribute to their approachability and acceptability for new immigrant populations and to adopt these in other primary care models.

The comparatively high percentage of recent immigrants who report need for a regular provider and the observed high percentage of these still using walk-in clinics and emergency rooms is also consistent with reported issues with accessing a regular provider among recent immigrants [8, 55]. These observed patterns among male and female recent immigrants underscore the barriers within the structure and delivery of primary care services. For example, whether health care services provide culturally responsive and safe health care including diversity, outreach services and language interpreter services may shape how and whether recent immigrants experience health care as approachable and acceptable. This is supported by research studies that find unmet need among recent immigrants based on how responsive services were, to their cultures, languages and beliefs [8, 56–59].

## Future Directions in Area of Study

Our findings suggest that sex shapes differences in patterns of health care seeking, reaching and utilization. Research that more accurately measures sex and gender will provide for more nuanced and improved examinations of patterns of access. Results also point to the importance of more research that adopts an intersectional lens to help understand experiences of health service use and perception of health among immigrant men.

## Limitations of the Study

On the CCHS, the variable “sex” is used with two categorical responses: “male” or “female.” Data pertaining to gender are not collected and we interpret this variable as capturing differences pertaining to both sex and gender. The cisnormative combination of sex and gender within this interpretation is a significant limitation of this analysis.

## New Contributions to Literature

Findings reveal associations between immigration, sex, and primary care access even after accounting for possible explanatory variables. Stratified comparisons allow for better identification of difficulties and gaps in access experienced by specific immigration and sex groups that are otherwise missed in international literature. Findings call into question the approachability and accessibility of primary care services, particularly for recent male immigrants. Remodeling primary care services to respond to diverse cultural and health care needs will help reduce disparities in access to primary care and may ultimately improve health outcomes among Canada’s immigrant communities. Internationally, the study suggests the need for further examination of the intersectional and gendered experiences of immigrants in accessing primary care in host countries in order to support responsive primary care policies.

## Data Availability

Data was accessed through Statistics Canada Public User Micro data files.
